# A data preprocessing strategy for metabolomics to reduce the mask effect in data analysis

**DOI:** 10.3389/fmolb.2015.00004

**Published:** 2015-02-02

**Authors:** Jun Yang, Xinjie Zhao, Xin Lu, Xiaohui Lin, Guowang Xu

**Affiliations:** ^1^Key Laboratory of Separation Science for Analytical Chemistry, Dalian Institute of Chemical Physics, Chinese Academy of SciencesDalian, China; ^2^Department of Entomology and Nematology, University of California, DavisDavis, CA, USA; ^3^School of Computer Science and Technology, Dalian University of TechnologyDalian, China

**Keywords:** metabolomics, data preprocessing, pattern recognition, biomarkers, differential metabolites

## Abstract

**Highlights**

- Developed a data preprocessing strategy to cope with missing values and mask effects in data analysis from high variation of abundant metabolites.
- A new method- ‘x-VAST’ was developed to amend the measurement deviation enlargement.
- Applying the above strategy, several low abundant masked differential metabolites were rescued.

Metabolomics is a booming research field. Its success highly relies on the discovery of differential metabolites by comparing different data sets (for example, patients vs. controls). One of the challenges is that differences of the low abundant metabolites between groups are often masked by the high variation of abundant metabolites. In order to solve this challenge, a novel data preprocessing strategy consisting of three steps was proposed in this study. In step 1, a ‘modified 80%’ rule was used to reduce effect of missing values; in step 2, unit-variance and Pareto scaling methods were used to reduce the mask effect from the abundant metabolites. In step 3, in order to fix the adverse effect of scaling, stability information of the variables deduced from intensity information and the class information, was used to assign suitable weights to the variables. When applying to an LC/MS based metabolomics dataset from chronic hepatitis B patients study and two simulated datasets, the mask effect was found to be partially eliminated and several new low abundant differential metabolites were rescued.

## Introduction

Metabolomics has been successfully applied in many fields including clinical research (Brindle et al., 2002; Yang et al., 2004, 2005; Abate-Shen and Shen, 2009; Sreekumar et al., 2009), drug discovery (Kell and Goodacre, 2014), toxicology (Keun, 2006; van Ravenzwaay et al., 2014), and phytochemistry (Fiehn, 2002; Mari et al., 2013). With the quantitative measure of the dynamic metabolic response of living systems to pathophysiological stimuli or genetic modification (Nicholson et al., 2002), the disease process and mechanism could be investigated in a synthesis induction way (Kell, 2004). Among the analytical technologies used in metabolomics, NMR (Pelczer, 2005; Wang et al., 2005; Pinto et al., 2014; Powers, 2014; Wagner et al., 2014; Worley and Powers, 2014), chromatography and their hyphenated techniques (Keun et al., 2003; Bijlsma et al., 2006; Craig et al., 2006; Dai et al., 2014; Peterson et al., 2014; Wachsmuth et al., 2014; Zhao et al., 2014) were the most popular.

In general, after samples are analyzed using various instruments, the data collected need be pre-processed including data alignment (Koh et al., 2010), normalization (Sysi-Aho et al., 2007) or internal standard correction, missing value correction, scaling and transformation (van den Berg et al., 2006; Enot et al., 2008; Veselkov et al., 2011; Want and Masson, 2011; Hrydziuszko and Viant, 2012; Kohl et al., 2012) before using various chemometrics methods (Trygg et al., 2007). A general strategy of data (pre-) processing and validation for human metabolomics studies was given by Bijlsma et al. (2006). However, they didn't describe how the data preprocessing method affects the results and what data preprocessing methods are to be selected for a given study.

Craig et al. (2006) investigated the scaling and normalization effects in details, two traditional scaling methods [mean centering and unit variance (Uv)] were compared using NMR data sets. It was concluded that mean centering (Ctr) could result in a parsimonious model, and Uv favored systematic changes with small variance while it confounds the potential useful information embedded in peak height and peak multiplicities. In another word, Uv may diminish the mask effect of the abundant metabolites, which is a common problem in proteomics and metabolomics fields. Unfortunately, at the same time, the deviations from measurements are significantly magnified since the measurement deviations are often higher at low concentrations, which will confound the results.

To eliminate the adverse effects of Uv mentioned above, several methods were developed. Keun et al. (2003) proposed a strategy for incorporating prior information into the scaling procedure called variable stability (VAST) scaling, in which each variable is assigned a weight according to its stability. Another method is orthogonal signal correction (OSC) (Wold et al., 1998). The OSC can extract the components with the maximum variance orthogonal to Y. This orthogonal model effectively filters obscuring variation in the data set. However, how many components should be retained appropriately becomes another challenge in the OSC procedure. Van den Berg et al. compared several different centering, scaling and transformations in a GC/MS data set and concluded that “the choice for a pretreatment method depends on the biological question to be answered” (van den Berg et al., 2006).

In the current study, we have developed a novel data preprocessing strategy to cope with the missing values and eliminate mask effects in data analysis from high variation of abundant metabolites. It consists of the following three steps: missing value correction, scaling and x-VAST. In the missing value correction step, a ‘modified 80% rule’ was proposed to cope with the missing value. In the scaling method, Pareto (User's Guide to SIMCA-P, 2005) was chosen to reduce the effect of the metabolite magnitude (i.e., eliminate the mask effect) without amplifying the measurement deviation too much. At last, a new method called as ‘x-VAST’ was developed to amend the measurement deviation enlargement after the VAST information and class information were used. The contour plots, which give an intuitionist view, were employed to illustrate the effects of each step. In order to test the developed data preprocessing strategy, the dataset from a metabolomics study of chronic hepatitis B patients was tested. Several masked differential metabolites were rescued. In addition, two simulated datasets were used to test if the proposed strategy could be generalized. The result indicated that the developed preprocessing strategy could improve the analysis of multivariate dataset of metabolomics by removing missing values and reducing mask effect.

## Materials and methods

### Plasma samples and high performance liquid chromatography-mass spectrometry (HPLC-MS) analysis

Thirty seven chronic hepatitis B patients hospitalized for acute deterioration in liver function and 50 healthy individuals were enrolled in this study. The detailed sample information and HPLC-MS analysis procedure were described in another paper (Yang et al., 2006). After peak alignment, 7347 ions were generated in the final reference peak list. The data set was an 87 × 7347 matrix. After preprocessed by missing value correction, scaling and x-VAST, partial least squares discriminant analysis (PLS-DA) was used to discovery the differential metabolites.

### Missing value correction

The data sets from the metabolic profiling analysis usually contain many zeros. They are considered as the missing value, which are artificial cutoffs from the peak alignment. The missing values could affect the correlation between variables, which would deteriorate the performance of multivariate analysis.

In order to reduce the number of zeros present, Smilde et al. applied a procedure referred as the ‘80% rule’ (Smilde et al., 2005). A variable will be kept if it has a non-zero value for at least 80% of all samples. One shortcoming is that some perfect differential metabolites might be lost according to the ‘80% rule’ when their concentrations were below the detect limitation in one specific class. In this work, the class information was utilized as the supervisor, the ‘80% rule’ was modified to a ‘variable is kept if the variable has a non-zero value for at least 80% in the samples of any one class’. In this paper, this new rule was called as ‘modified 80% rule’.

### Scaling methods

In the scaling section, Ctr, Uv, Pareto and logarithm (*ln*) transformation were compared in diminishing the mask effects and finding the differential metabolites more efficiently. To avoid the confusion, we adopt the following definitions as in the SIMCA-P manual (User's Guide to SIMCA-P, 2005).

*Mean centering (Ctr):*

(1) $${x^{\prime }}_{ik}=x_{ik}-{\bar{x}}_{k}$$

Where *x*′_*ik*_ is the value after scaling, *x*_*ik*_ is the original value; *x*_*k*_ is the mean of the variable *k*.

*Uv:*

(2) $${x^{\prime }}_{ik}=\frac{x_{ik}}{s_{k}}$$

Where *s*_*k*_ is the standard deviation of the variable *k*.

*Pareto:*

(3) $${x^{\prime }}_{ik}=\frac{x_{ik}}{\sqrt{s_{k}}}$$

*ln transformation:*

(4) $${x^{\prime }}_{ik}=\text{ln }x_{ik}$$

Here, we propose a new supervised scaling method based on VAST method, which is referred as ‘x-VAST’. And VAST, supervised VAST methods (Keun et al., 2003) are employed for comparison.

*x-VAST:*

(5) $${x^{\prime }}_{ik}=\text{max}\left(\frac{{\bar{x}}_{1k}}{s_{1k}},\frac{{\bar{x}}_{2k}}{s_{2k}},\frac{{\bar{x}}_{3k}}{s_{3k}}\ldots \frac{{\bar{x}}_{jk}}{s_{jk}}\ldots \frac{{\bar{x}}_{nk}}{s_{nk}}\right)•x_{ik}$$

Here, *x*_*jk*_ and *s*_*jk*_ are the mean and standard deviation of the variable *k* for the *j*th class, respectively, and n is the total number of classes.

*VAST:*

(6) $${x^{\prime }}_{ik}=\frac{{\bar{x}}_{k}}{s_{k}}•x_{ik}$$

supervised VAST (s-VAST):

(7) $${x^{\prime }}_{ik}=\left(\frac{1}{n}\sum_{j=1}^{n}\frac{{\bar{x}}_{jk}}{s_{jk}}\right)•x_{ik}$$

The preprocessing methods mentioned above were all realized in self-developed scripts written in MATLAB software (Mathworks, Natick, MA).

### Contour plot and PLS-DA

The contour plot was employed to visualize the data. In the plot, x-coordinate is corresponding to the variables, y-coordinate is corresponding to the samples. The plot is straightforward to show difference among the effect from different data preprocessing methods.

To compare the final classification results and find the differential metabolites, PLS-DA in SIMCA-P software (Umetrics, Sweden) was employed.

### Validation with simulated dataset

In order to test if the proposed method could be generic, two datasets [one includes 140 variables, another includes 1400 variables; both includes two class of samples (*n* = 20 in each class)] were generated to validate it.

The smaller dataset (variable number is 140) including 50 high abundant random variables (HNM variables), 50 low abundant random variables (LNM variables), 10 high abundant and big change variables with 10 times difference on average (HGM variables), 10 high abundant and medium change variables with three times difference on average (HMM variables), 10 low abundant and big change variables with 10 times difference on average (LGM variables), 10 low abundant and medium change variables with three times difference on average (LMM variables). The bigger dataset includes similar setup but has 10 times more variables. The detail codes for generating the simulated datasets are included in the Supplementary File for information. In brief, random normal distribution function was used to generate each group variables with different abundance and variations as shown in the code.

## Results and discussion

### Missing value correction

**Figure 1:**
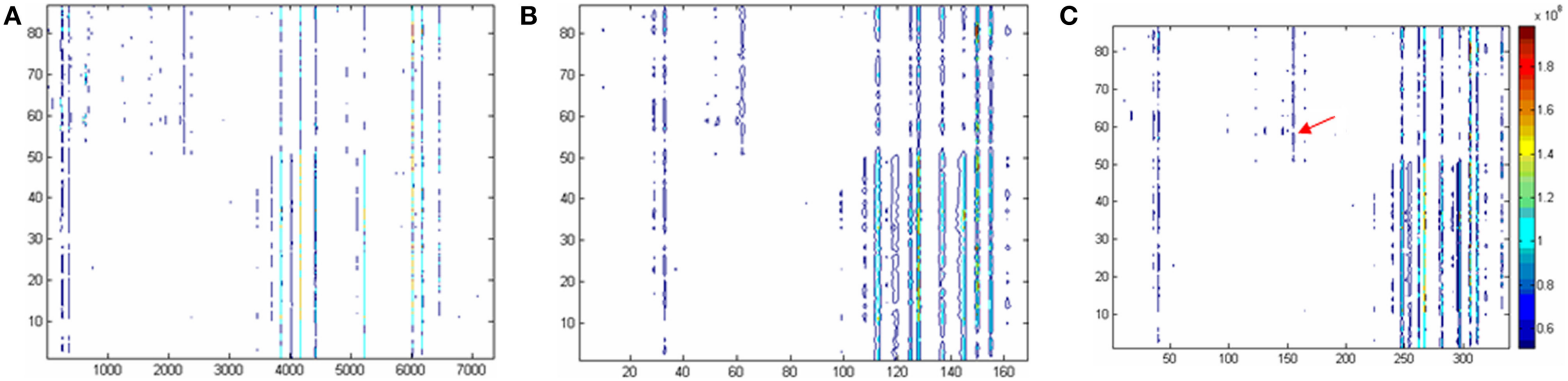
**Two dimensional contour plots based on (A) the raw data, (B) the data excluding missing values according to 80% criteria, and (C) modified 80% criteria.** The horizontal coordinate is corresponding to the variable No. The longitudinal coordinate is corresponding to the sample No. And the color is corresponding to the responses of the variables. To be convenient, the variables in original data were named as var +“_”+ number like var_1, the variables in **Panel C** (i.e., the raw data were corrected by modified 80% rule) were expressed as VAR +“_”+number, such as VAR_1.

**Figure 2:**
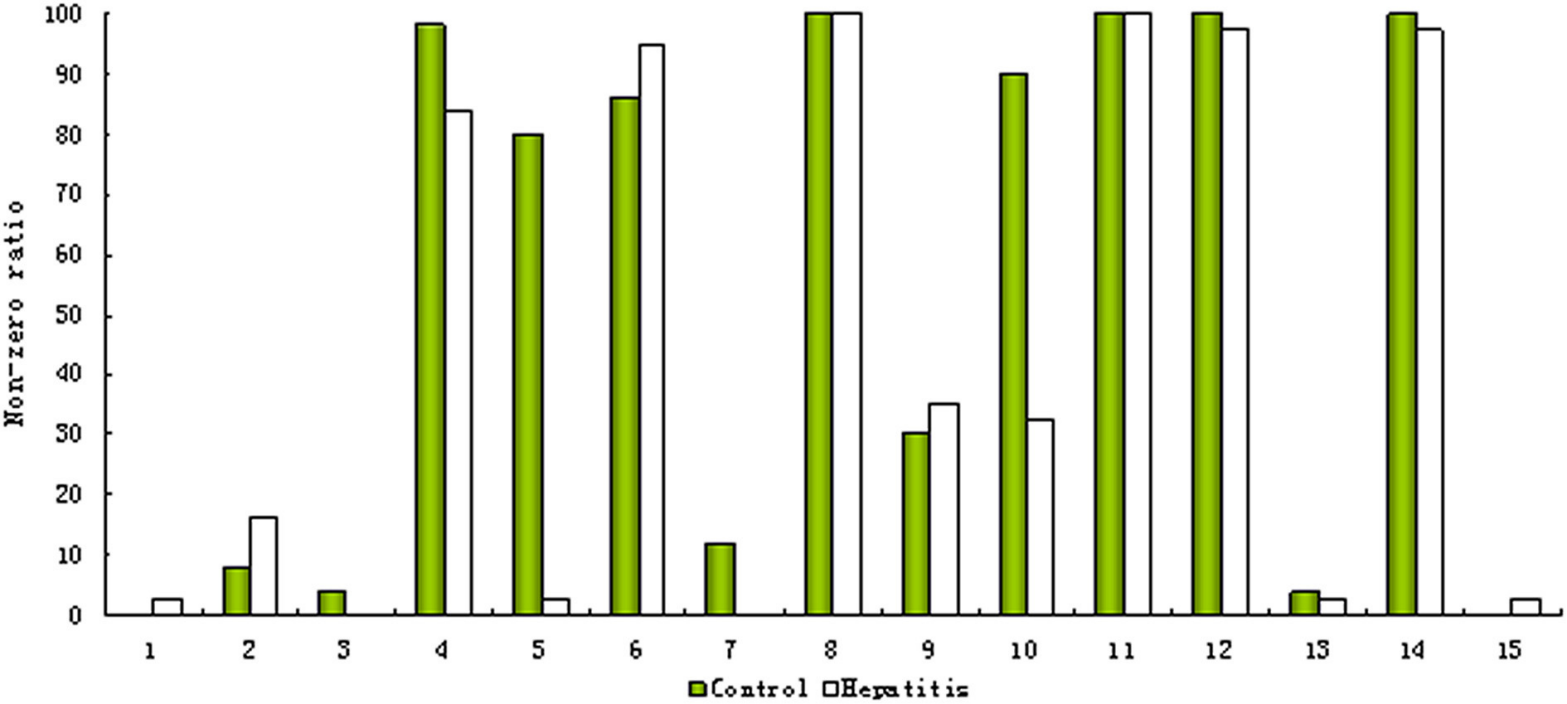
**Non-zero ratios in the control and hepatitis groups of the first 15 ions**.

As mentioned above, the ‘80% rule’ is often followed when missing values are present in the data set. Figures 1A,B shows the contour plots of the raw data and the data corrected according to the ‘80% rule’. After corrected, the variable number was reduced dramatically, most of them were deleted and only 169 were reserved. As illuminated in the following section, in this step some useful differential metabolites were also deleted. As an example, Figure 2 shows the non-zero ratio of the first 15 variables of the raw data in each class sample (control and hepatitis). From the figure, the variables can be divided into three types:

1. Type 1, which values in most of the samples in each class is zero such as var_1, var_2, and var_3, it indicates that these variables have a very low concentration, and present method can't correctly measure them and should be deleted.
2. Type 2, which values in most of the samples are zero in one class or several classes, but in the samples of the remaining at least one class most of them are non-zero, such as var_5. These variables are perfect biomarkers which can accurately differentiate different groups. The variables of this type should be reserved instead of being deleted.
3. Type 3, which values in most of the samples in each class are non-zero such as var_8, var_11, var_12, and var_14, it indicates that the value of this type variation could be measured and should be reserved.

In current study, a ‘modified 80% rule’, is suggested: the variables which non-zero values in any class of the samples are above 80% should be reserved. According to this rule, the type 2 variables defined above will be rescued. Figure 1C gives the contour plot processed according to the ‘modified 80% rule’. Compared to 80% rule, many type 2 variables were rescued (170 out of 339 are new). As an example, it can be found that VAR_165 is present according to the ‘modified 80% rule’ but absent according to the ‘80% rule’ (see arrow position in Figure 1C). The significant difference is found when *t*-test is applied to this variable. It could be concluded that the ‘modified 80% rule’ saves more differential metabolites (around two times more).

### Mask effects and various scaling methods

When the average responses of the 7347 ions were compared, the dynamic range (minimum to maximum ratio) of these ions is 3.22 × 10^−5^. It resulted in the fact that the variable with high responses would be endowed with a bigger weight and their variations have dominant impacts on the result if no scaling methods were employed. The minor peaks will be masked by the major ones or noise although their biology meaning may be of importance.

The mask effect could be eliminated, at least partly reduced if the variables were divided by their deviations, i.e., scaling according to Uv. Each new variable would have identical weight for the identical variance i.e., Uv. The height information was discarded while only the deviation information was reserved. It seems that Uv is an ideal scaling method to eliminate the mask effects and perfectly suit for metabolomics application to differential metabolite discovery if all variables could be accurately measured and the deviation from measurement could be ignored. Unfortunately, it is not always true especially when the metabolite responses are near the detection limit. The measurement deviation would account for the major part in the deviation information when the peaks were just above the detection limit. In other words, Uv scaling method magnifies the measurement variations for the low abundance metabolites. In this situation, the peak response information still gives some information about how much probability the deviation from measurement should be considered. In another word, the peak information should be reserved to some extent.

**Figure 3:**
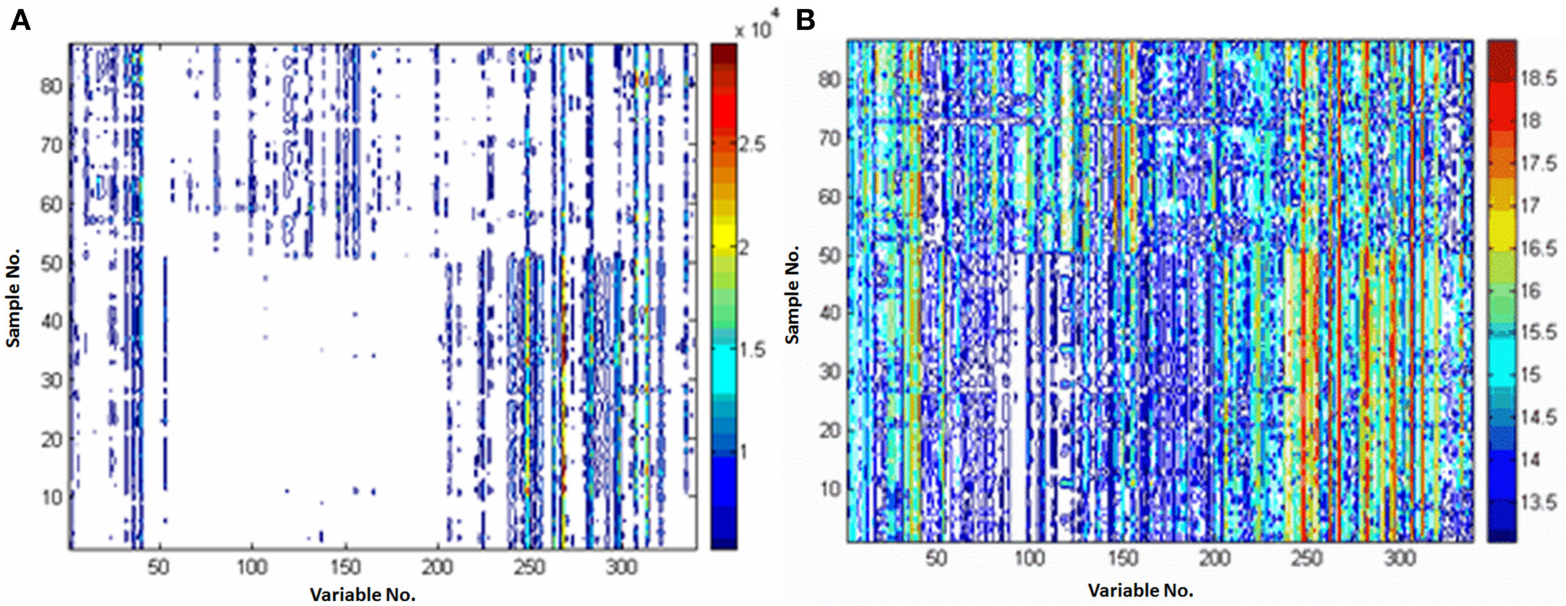
**Comparison of the different preprocessing methods. (A)** preprocessed with Pareto; **(B)** preprocessed with *ln* transformation. X-axis is variable number and y-axis is sample number.

Pareto and *ln* transformation could satisfy the requirement. Figure 3 shows the contour plots scaled by the Pareto or *ln* transformation. Compared with the raw data without scaling (Figure 1C), it could be found that the response information was reserved too little to discover the differential metabolites after the *ln* transformation (Figure 3B), the Pareto scaling seems a good compromise between diminishing mask effects and avoiding magnifying the measurement deviation of low concentration metabolites (Figure 3A).

### x-VAST

To solve the dilemma mentioned above, many algorithms were developed. Keun et al. (2003) thought the VAST will improve the analysis of any multivariate dataset where group differences were significantly obscured by other variation. Here, x-VAST was developed to amend the adverse effect mentioned above after scaling. As comparison, the VAST and s-VAST were also employed to utilize the VAST to adjust the variables' weights. In general, the variables, which variation was mainly from measurement deviation or from the individual variation, have lower stability (smaller *x*/*s* value). It could be expected that the combination of the VAST and scaling methods mentioned above could diminish the mask effects with fewer side effect.

**Figure 4:**
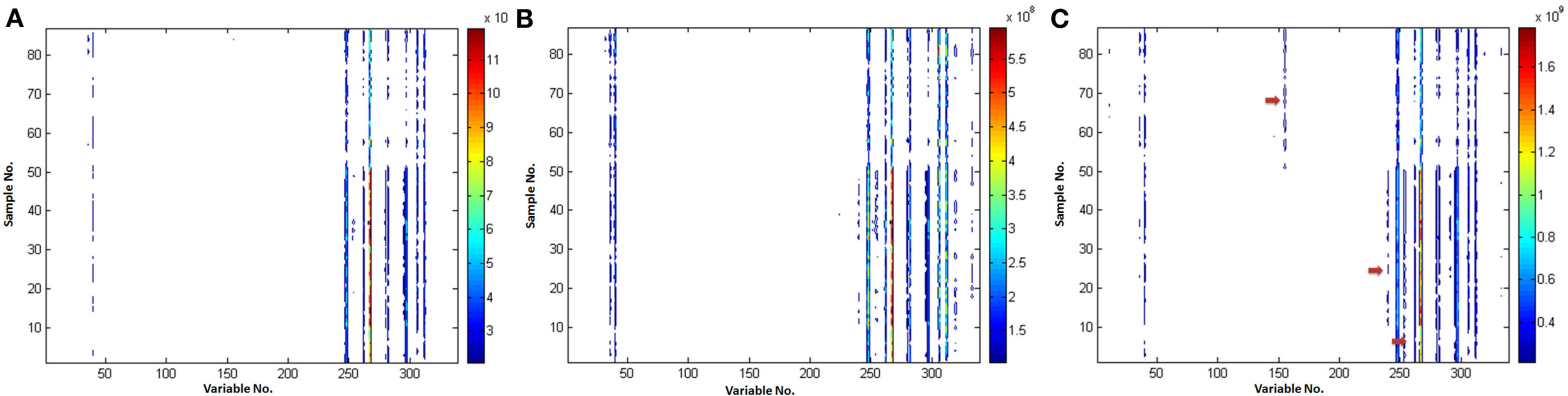
**Comparison of the different VAST scaling methods. (A)** VAST; **(B)** s-VAST; **(C)** x-VAST. Red arrow indicates the specially enhanced variables processed after x-VAST' method. X-axis is variable number and y-axis is sample number.

Comparison of the various VAST scaling methods is shown in Figure 4. It could be found that (i) the noise was eliminated and the stability of variables was enhanced after scaled by all of the VAST methods; (ii) the variables (e.g., VAR_60, VAR_106, the red arrows) which have distinct different values in the two classes, got a larger weights after scaled by x-VAST, while the difference of these variables was not found by the VAST and s-VAST. Confirmed by the following PLS-DA result, these two variables had prominent contribution to the classification.

It could be concluded that the variables, which have stable values in one class while unstable values near detection limit in another class, would be assigned to a smaller weights in VAST and s-VAST. In fact, these variables are the most useful biomarkers, they should be assigned to the maximum weights, which was the case in x-VAST.

### PLS-DA analyses

**Figure 5:**
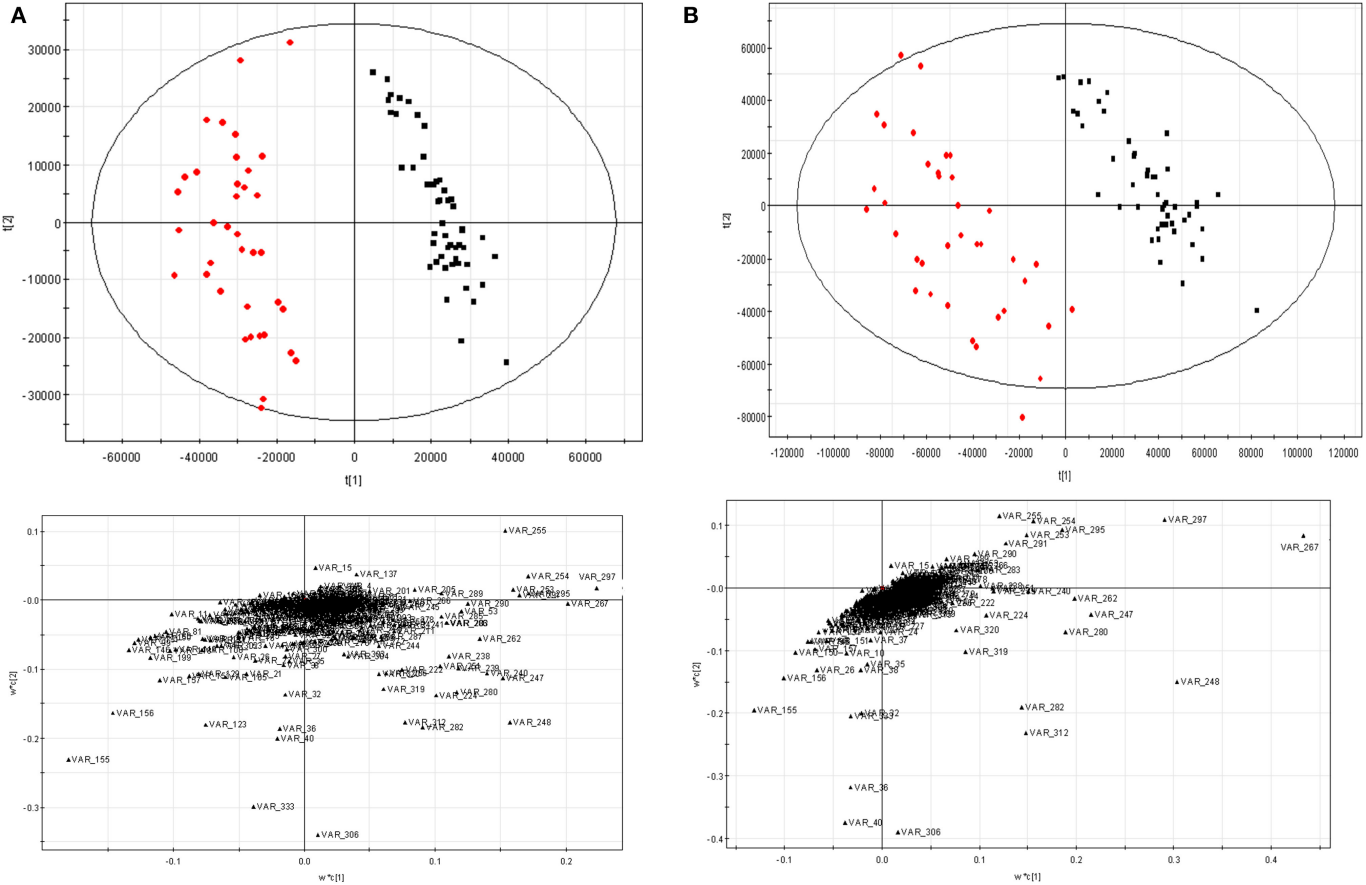
**PLS-DA results scaled by Pareto-Ctr and Pareto-x-VAST-Ctr. (A)** Pareto-Ctr; **(B)** Pareto-x-Vast-Ctr. Left, score figure. □ hepatitis, ■ control. Right, loading figure.

PLS-DA was employed as another way to assess the data preprocessing strategy mentioned above. The data scaled by 11 scaling methods were fed to PLS-DA, respectively. The results were given in the Supplementary Materials (Table S1, Figure S1). Here, only the score and loading plots scaled by Pareto-Ctr and Pareto-x-VAST-Ctr are given in Figure 5. After scaled by x-VAST, A group of variables were recognized as highly important metabolites (e.g., VAR_267, VAR_248, VAR_297, VAR_36, VAR_40) became more important, while other variables (e.g., VAR_333) became less important.

The comparison of new differential metabolites and old ones (the first 20 differential metabolites) was given in Table 1, five new differential metabolites (var_359, var_369, var_3703, var_3705, var_3866) were identified instead of five old differential metabolites (var_644, var_686, var_741, var_4178, var_6461). In these deleted old differential metabolites, four of them (var_644, var_686, var_741, var_4178) were found having too many missing values. The last one, var_6461, which is corresponding to VAR_333 in Figure 1C, failed in the *t*-test.

**Table 1:** **Using the developed data preprocessing strategy, several differential metabolites were rediscovered**.

**Before preprocessed**				**After preprocessed**			
**var__ID**	**retention time (min)**	**m/z**	**Identification result**	**var_ID**	**retention time (min)**	**m/z**	**Identification result**
var_5229	17.22	524.5	LPC C18:0	var_4177	15.33	496.5	LPC C16:0
var_4177	15.33	496.5	LPC C16:0	var_3850	14.75	520.5	LPC C18:2
var_4167	15.25	478.2	LPC C16:0 Fragment	var_5229	17.22	524.5	LPC C18:0
var_5226	17.21	506.6	LPC C18:0 Fragment	var_3849	14.75	502.5	LPC C18:2 fragment
var_3850	14.75	520.5	LPC C18:2	var_4167	15.25	478.2	LPC C16:0 fragment
**var_686 ^a^**	7.78	235.2	UN^a^	var_4417	15.84	504.4	LPC C18:1 fragment
**var_644 ^a^**	7.55	235.2	UN^a^	var_6169	19.81	282.4	
var_4422	15.85	522.4	LPC C18:1	var_5226	17.21	506.6	LPC C18:0 fragment
var_3849	14.75	502.5	LPC C18:2 Fragment	var_4422	15.85	522.4	LPC C18:1
var_2266	12.34	414.2	GCDCA or GDCA Fragment	var_6014	19.49	256.4	UN
var_6014	19.49	256.4	UN^a^	var_4022	15.05	478.4	LPC C16:0 fragment
var_6169	19.81	282.4	UN^a^	**var_369 ^b^**	5.87	188.2	Trp fragment
**var_6461**	21.06	284.3	UN^a^	**var_3705 ^b^**	14.52	520.3	LPC C18:2
var_4417	15.84	504.4	LPC C18:1 Fragment	var_4021	15.05	184.2	Phosphatidylcholine moiety of LPC C16:0
var_4022	15.05	478.4	Fragment of LPC C16:0	var_2266	12.34	414.2	GCDCA or GDCA Fragment
var_4024	15.05	496.1	LPC C16:0	var_4024	15.05	496.1	LPC C16:0
var_4021	15.05	184.2	Phosphatidylcholine moiety of LPC C16:0	**var_359 ^b^**	5.86	146.1	Trp fragment
var_5104	16.89	524.4	LPC C18:0	var_5104	16.89	524.4	LPC C18:0
**va _4178 ^a^**	15.34	479.3	Isotope of 478.4	**var_3866 ^b^**	14.8	544.3	LPC C18:3
**var_741 ^a^**	7.99	235.3	UN^a^	**var_3703 ^b^**	14.52	502.3	LPC C18:2 fragment

*The following table compared the differential metabolites defined by PLS-DA before and after using developed preprocessing strategy*.

*The variables in bold font highlighted the different markers before and after the preprocessing strategy used*.

a *Deleted differential metabolites after preprocessed*.

b *Newly found differential metabolites after preprocessed*.

In the newly found differential metabolites list, two of them (var_369 and var_359) were tryptophan fragments according to authentic standard sample run under the same conditions. Tryptophan is an essential amino acid, a constituent of proteins. In addition, tryptophan is also a substrate for two important biosynthetic pathways: tryptophan 5-hydroxylase pathway to generate neurotransmitter 5-hydroxytryptamine (serotonin); and the formation of kynurenine derivatives and nicotinamide adenine dinucleotides. In addition, it was reported that tryptophan catabolites are prognostic biomarkers for the severity of chronic liver diseases in potential transplant recipients (Lahdou et al., 2011).

The other three (var_3703, var_3705, var_3866) were identified as lysophosphatidylcholines (LPCs). LPCs regulate many biological processes including cell proliferation, inflammation and tumor cell invasiveness. LPCs promotes inflammatory by expressing endothelial cell adhesion molecules and growth factors, monocyte chemotaxis, and activating macrophage.

### Validation of x-VAST with simulated datasets

In order to validate the proposed method, two simulated datasets were generated as method section described. The datasets were fed to SIMCA-P for the followed multivariate data analyses. The VIP (Yang et al., 2006) order was chose to reflect how these variables ranked as potential markers. Tables 2, 3 showed the comparison of markers identified by PLS-DA using the original datasets, the dataset with VAST and x-VAST treated.

**Table 2:** **Rank of markers by PLS-DA using small simulated dataset (140 variables) preprocessed by none, VAST and x-VAST**.

**Variable groups**	**Rank 1–10**	**Rank 11–20**	**Rank 21–30**	**Rank 31–40**
HG004D (var101-110)	7	2	1	0
HMM (var111-120)	3	6	1	0
LGM (var121-130)	0	1	7	2
LMM (var131-140)	0	1	1	4
**PREPROCESSED BY VAST**				
HGM (var101-110)	7	2	1	0
HMM (var111-120)	3	5	0	0
LGM (var121-130)	0	1	1	4
LMM (var131-140)	0	0	0	4
**PREPROCESSED BY x-VAST**				
HGM (var101-110)	7	2	1	0
HMM (var111-120)	3	6	1	0
LGM (var121-130)	0	2	6	2
LMM (var131-140)	0	0	1	2

**Table 3:** **Rank of markers by PLS-DA using big simulated dataset (1400 variables) preprocessed by none, VAST and x-VAST**.

**Variable groups**	**Rank 1–100**	**Rank 101–200**	**Rank 201–300**	**Rank 301–400**
HGM (var1001-1100)	67	30	1	2
HMM (var1101-1200)	22	33	33	7
LGM (var1201-1300)	11	37	39	9
LMM (var1301-1400)	0	0	27	54
**PREPROCESSED BY VAST**				
HGM (var1001-1100)	61	28	1	2
HMM (var1101-1200)	18	33	33	7
LGM (var1201-1300)	9	38	38	8
LMM (var1301-1400)	0	0	25	48
**PREPROCESSED BY x-VAST**				
HGM (var1001-1100)	62	33	2	2
HMM (var1101-1200)	18	33	34	10
LGM (var1201-1300)	7	33	44	9
LMM (var1301-1400)	0	0	18	53

The concept behind VAST and x-VAST is to increase the rank for stable (high abundant, low variation) variables and decrease the rank for unstable (low abundant, high variation) variables. So, the rank for HMM variables, which have high abundance and lower relative variation, will move toward the beginning; the rank for LMM variables, which have low abundance and higher relative variation, will move toward the end of VIP lists. In both tables, the LMM variables did move toward to the lower rank when preprocessed by VAST and x-VAST.

**Figure 6:**
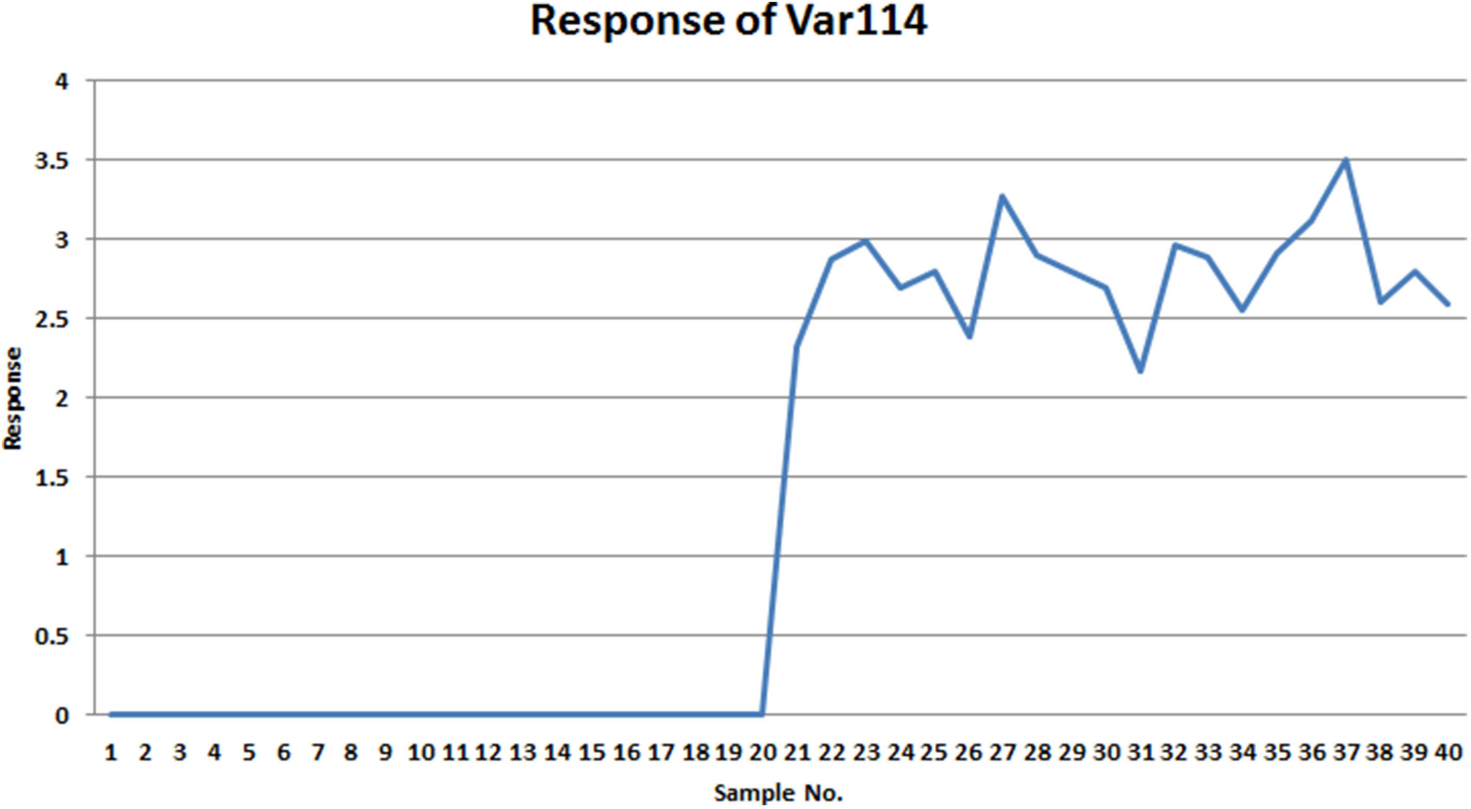
**The response of var114 in small simulated dataset.** It clearly shows that this variable is a good marker to differentiate two groups.

Comparing VAST and x-VAST, there are more markers were kept by x-VAST. For example, in Table 2, there is more markers identified in HMM groups. Figure 6 shows an example of the new identified biomarker (var 114). It clearly shows that, the responses of the var 114 are low abundant in one class. The preprocess of VAST did not identify this variable as biomarker because of the bigger variation from two classes. On the contrary, the preprocess of x-VAST can pick up this difference and identified this biomarker. The scenario of Var114 is just like what we saw in the real metabolomics dataset mentioned above.

The biggest difference between VAST and x-VAST was found for variables in LGM group, which has low abundance and bigger difference between two classes. As both Tables 2, 3 shown, VAST removed many markers because of low stability (average/variation) for these variables inspite of big difference between two classes. On the contrary, x-VAST used the higher stability calculation (average/variation) in one class as the weight for the variables. Then, more variables in this group were rescued back in biomarker list.

## Conclusions

The data preprocessing is a critical step in information mining of metabolomics studies, it directly influences the discovery of differential biomarkers. In this work, the missing values and the relationship between mask effect and scaling methods were studied. An optimal strategy including a ‘modified 80% rule’, Pareto scaling and x-VAST was suggested. When a dataset from acute deterioration in liver function of chronic hepatitis B was fed to the suggested strategy, several new differential metabolites masked by noise or other big peaks were rediscovered. Furthermore, two simulated datasets were used to test proposed method. It was shown that some masked marker was rescued by x-VAST. In the future, we will test it in another separate study to assess how useful this strategy is in a general metabolomics study. Although we use HPLC-MS dataset as a test dataset, it should be noted that the strategy could be used in other metabolomics research and other omics' datasets from different analytical platforms.

### Conflict of interest statement

The authors declare that the research was conducted in the absence of any commercial or financial relationships that could be construed as a potential conflict of interest.
