# Supplementary material for: A data preprocessing strategy for metabolomics to reduce the mask effect in data analysis
Source: Front Mol Biosci. 2015 Feb 2;2:4. doi: 10.3389/fmolb.2015.00004 (PMC4428451; doi:10.3389/fmolb.2015.00004)
Supplement: Supplementary file 1 [file Table1.DOCX]

**Supplementary materials**

Table S1 PLS-DA model parameter comparison of several preprocessing methods

|  | Preprocessing method | A* | R^2^X | R^2^Y | Q^2^ |
| --- | --- | --- | --- | --- | --- |
| 1 | none | 3 | 0.953 | 0.95 | 0.944 |
| 2 | Ctr | 3 | 0.763 | 0.963 | 0.95 |
| 3 | Uv-Ctr | 4 | 0.598 | 0.978 | 0.944 |
| 4 | Pareto-Ctr | 3 | 0.653 | 0.972 | 0.943 |
| 5 | ln transformation | 2 | 0．512 | 0．977 | 0．959 |
| 6 | x-VAST | 3 | 0.983 | 0.951 | 0.942 |
| 7 | x-VAST -Ctr | 3 | 0.873 | 0.962 | 0.951 |
| 8 | x-VAST -Uv | 4 | 0.598 | 0.978 | 0.944 |
| 9 | x-VAST –Pareto-Ctr | 3 | 0.727 | 0.970 | 0.949 |
| 10 | Uv-Ctr- x-VAST | 4 | 0.598 | 0.978 | 0.944 |
| 11 | Pareto- x-VAST | 4 | 0.963 | 0.965 | 0.943 |
| 12 | Pareto- x-VAST -Ctr | 4 | 0.751 | 0.969 | 0.948 |

* A: principal component number.


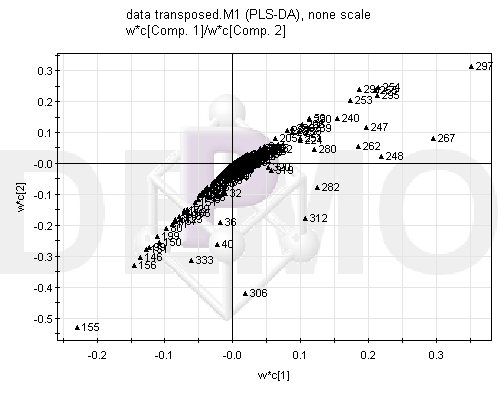

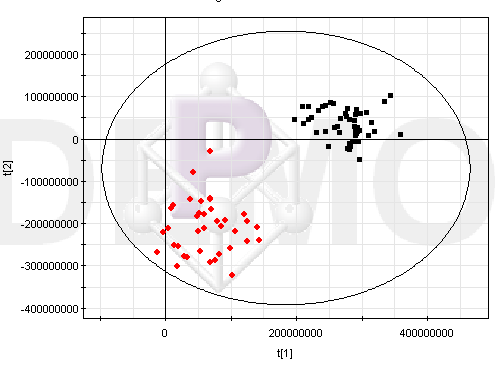


a) none


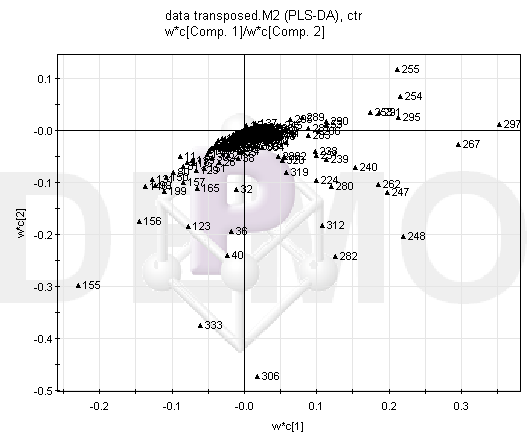

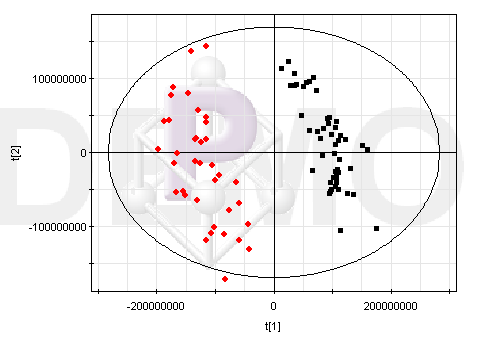


b) Ctr


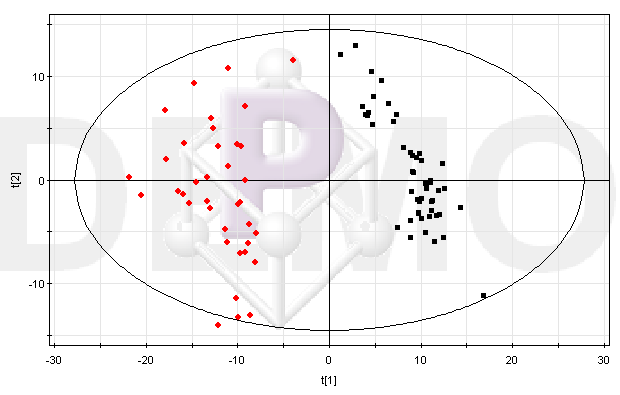

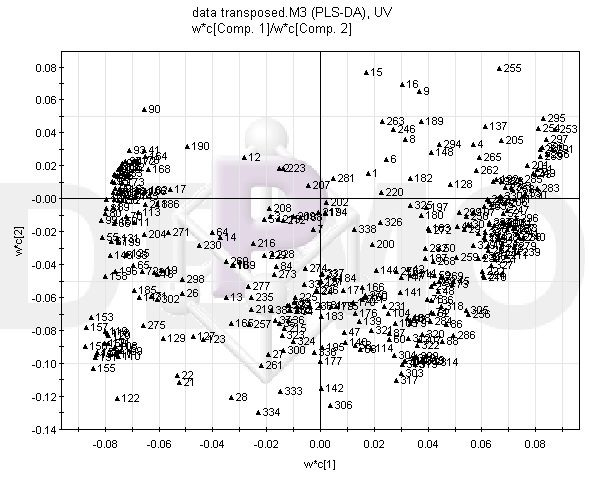


c) Uv－Ctr


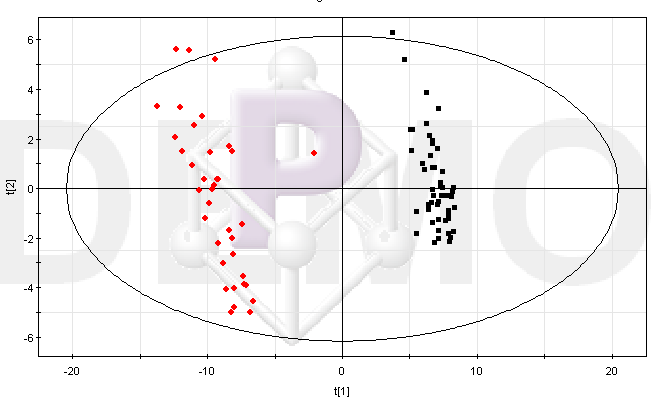

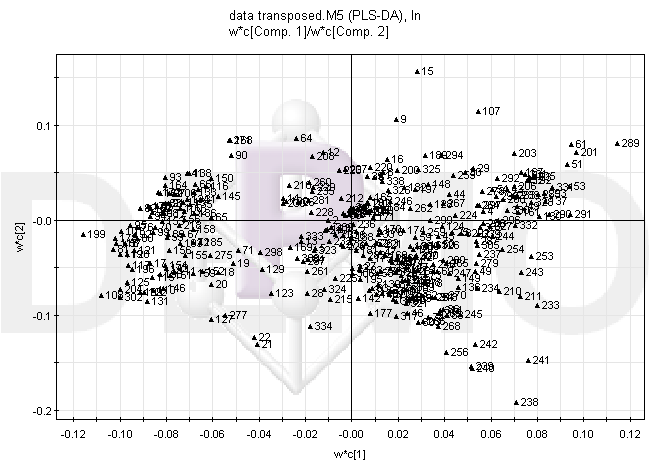


d) ln transformation


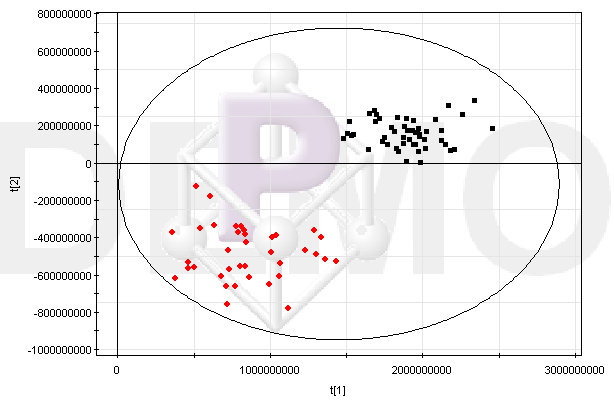

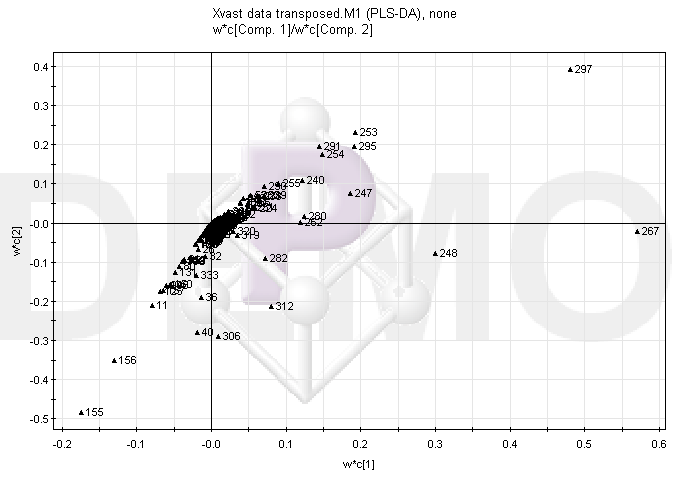


e) x-VAST


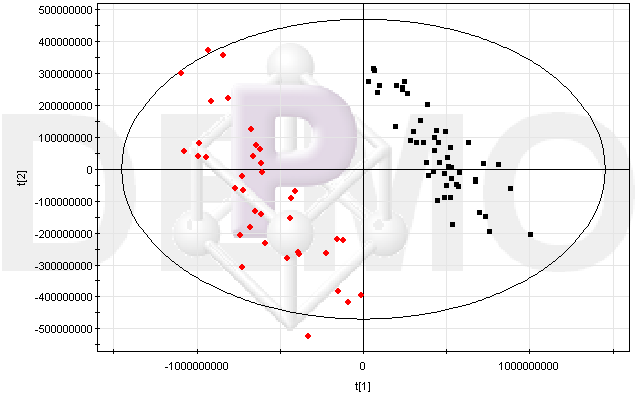

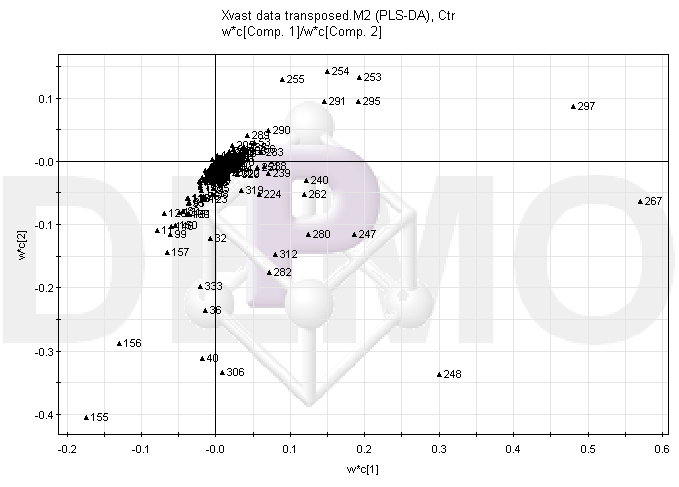


f) x-VAST ctr


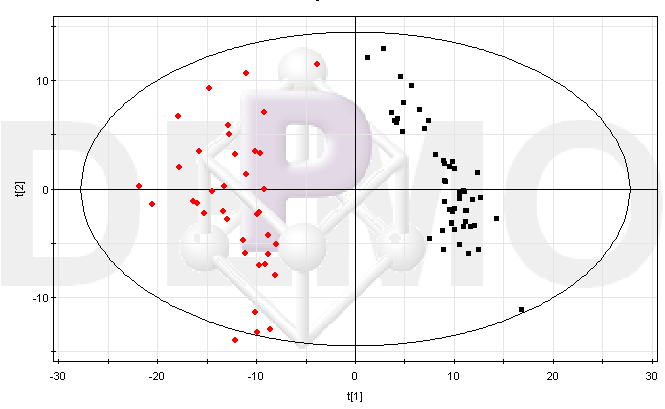

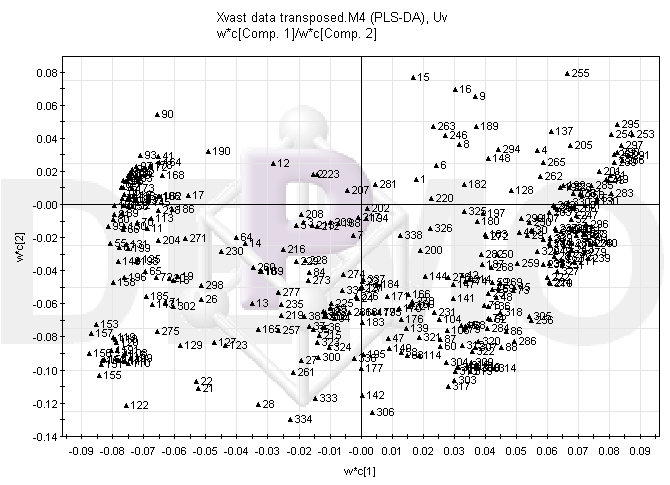


g) x-VAST-Uv


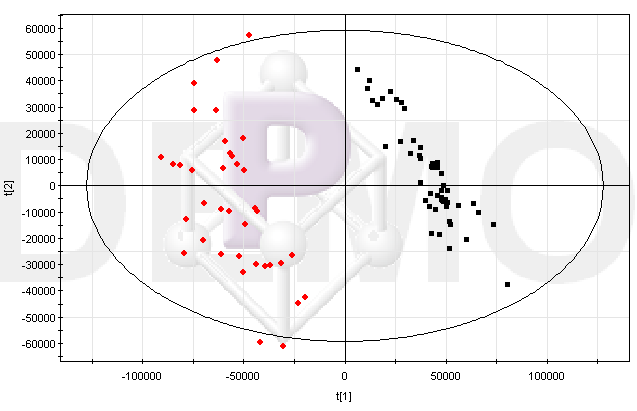

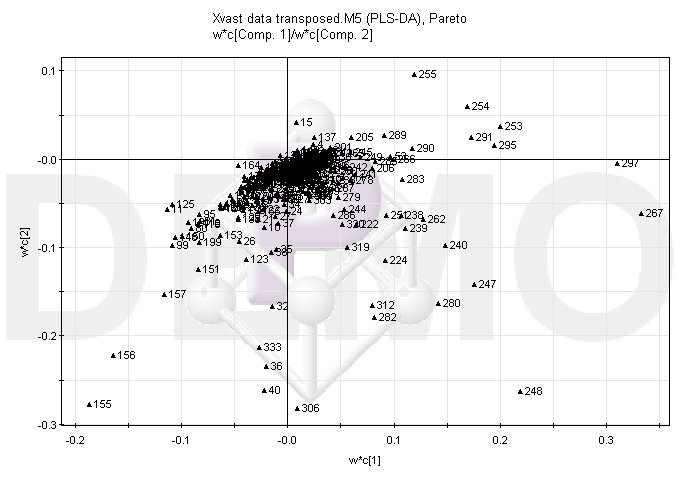


h) x-VAST-Pareto-Ctr


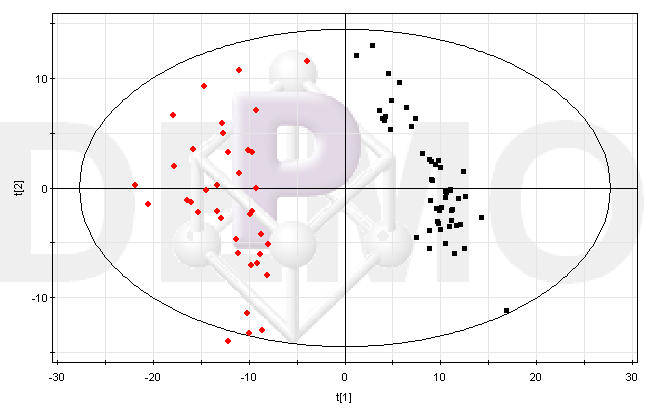

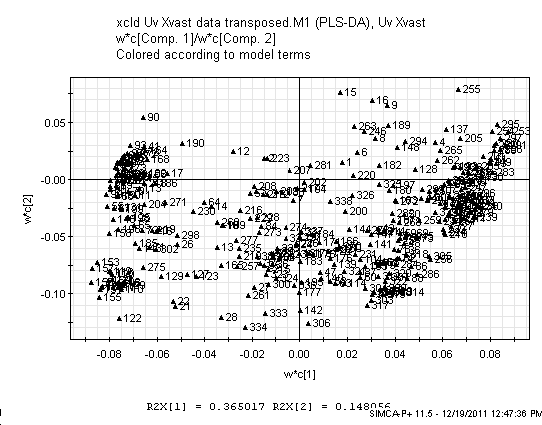


i) Uv-Ctr x-VAST


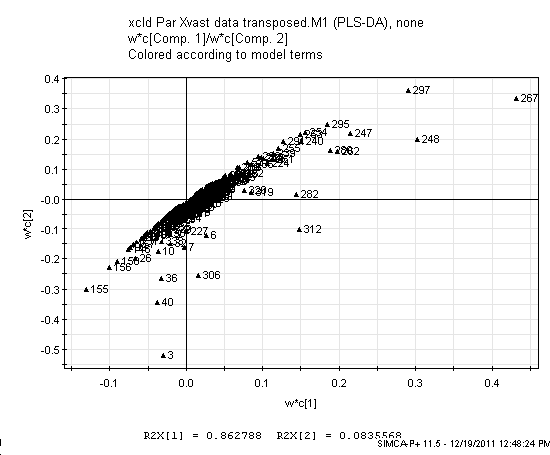

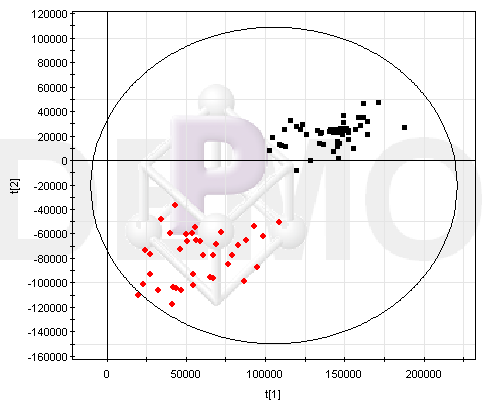


j) Pareto-x-VAST

Fig. S1. PLS-DA results after various preprocessing methods. All the labels in the loading plots are corresponding to the variables corrected by modified 80% rule. For example, 267 in loading plot of j) means VAR_267. （■ control，◆ hepatitis）
