# Supplementary figures and images for: A data preprocessing strategy for metabolomics to reduce the mask effect in data analysis
Source: Front Mol Biosci. 2015 Feb 2;2:4. doi: 10.3389/fmolb.2015.00004 (PMC4428451; doi:10.3389/fmolb.2015.00004)

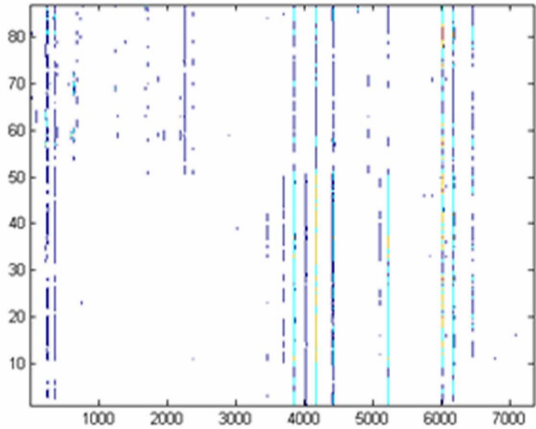

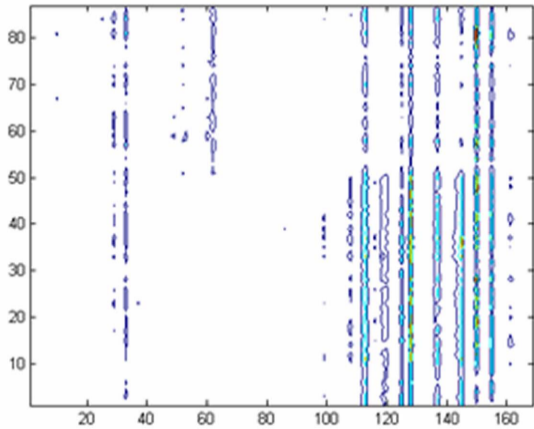

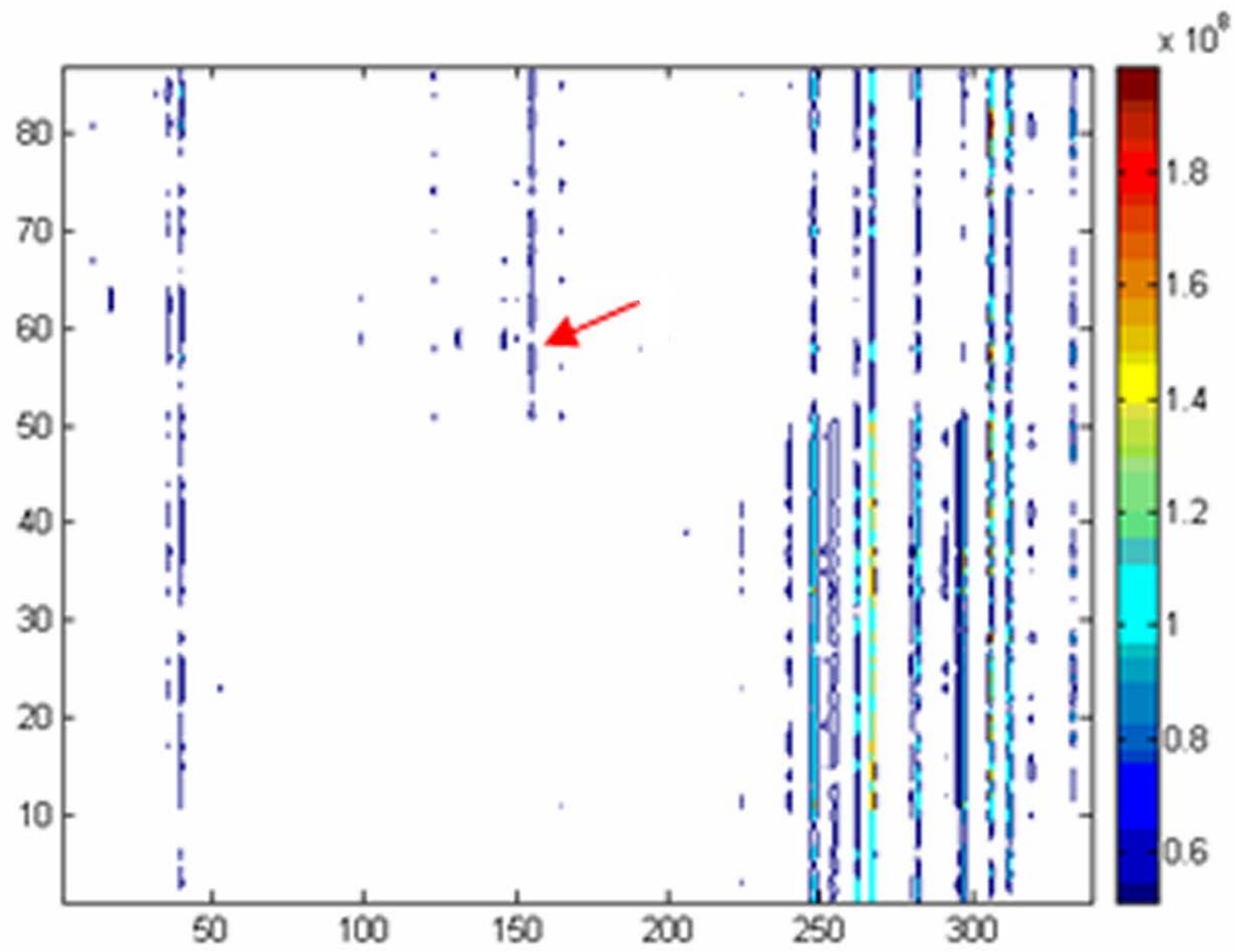

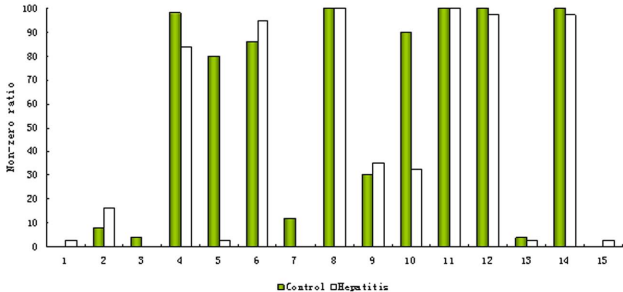

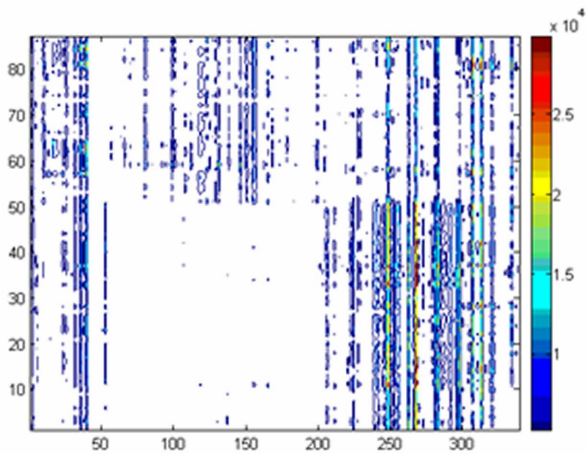

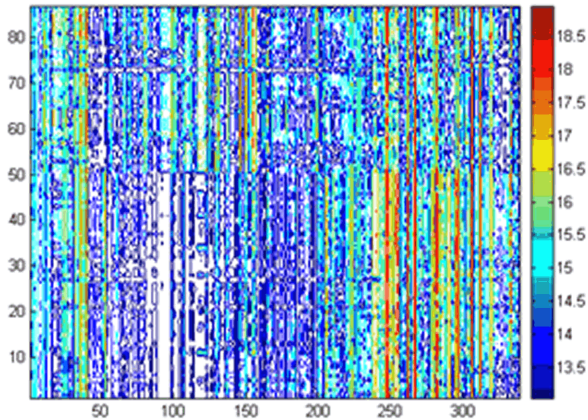

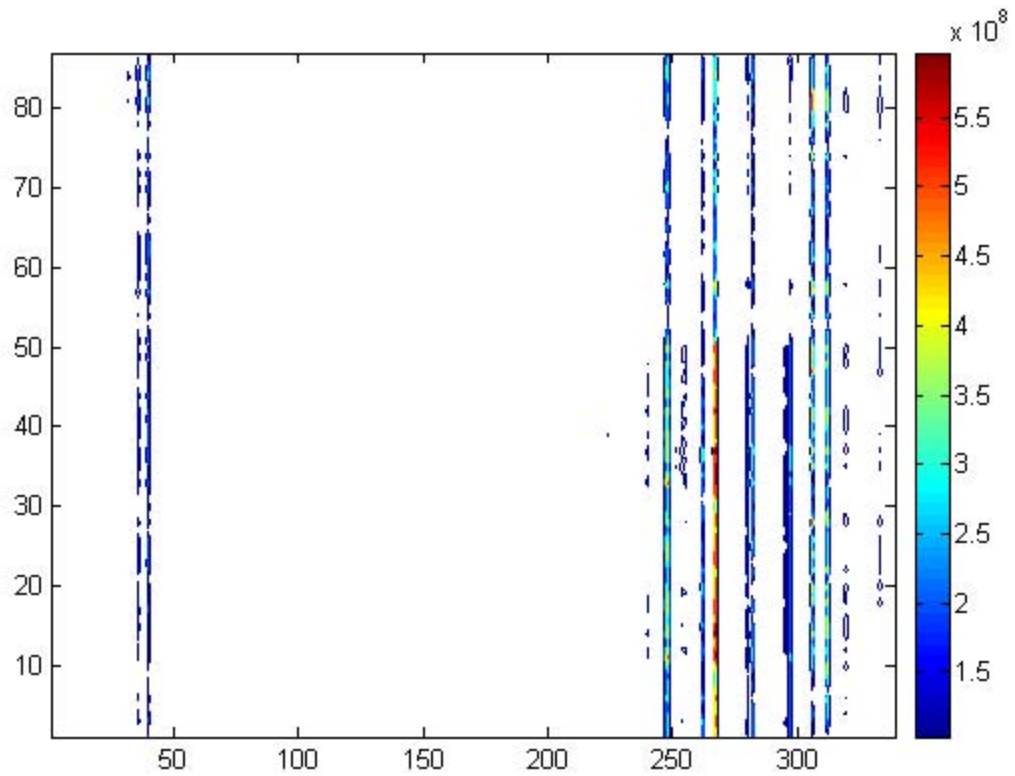

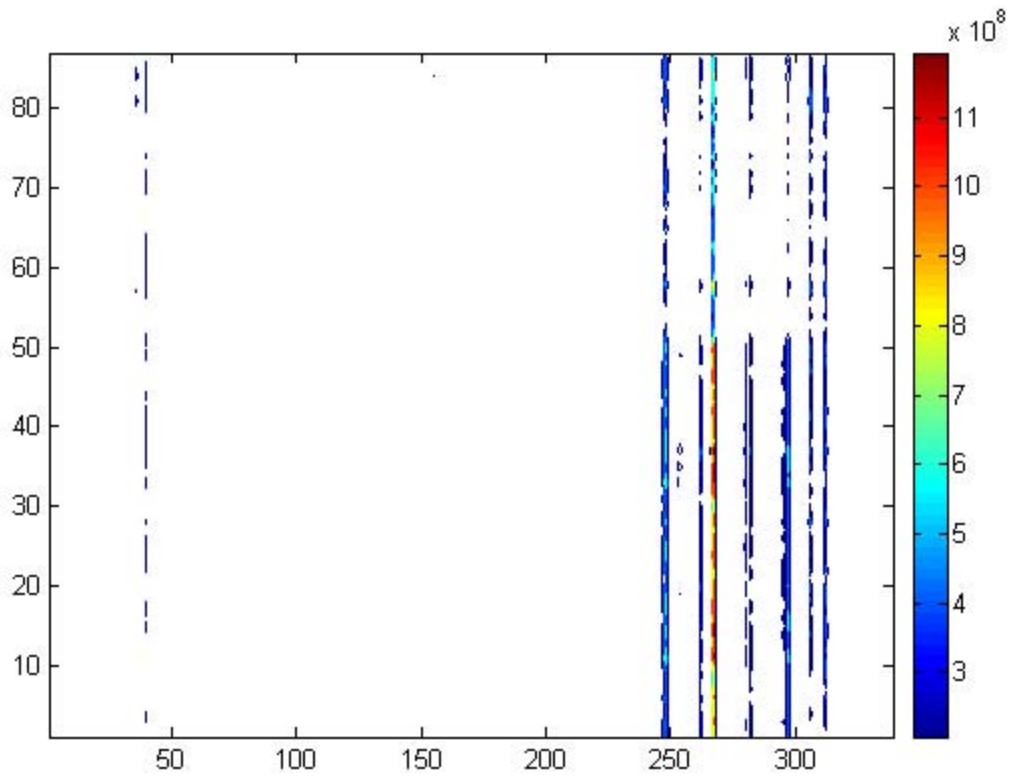

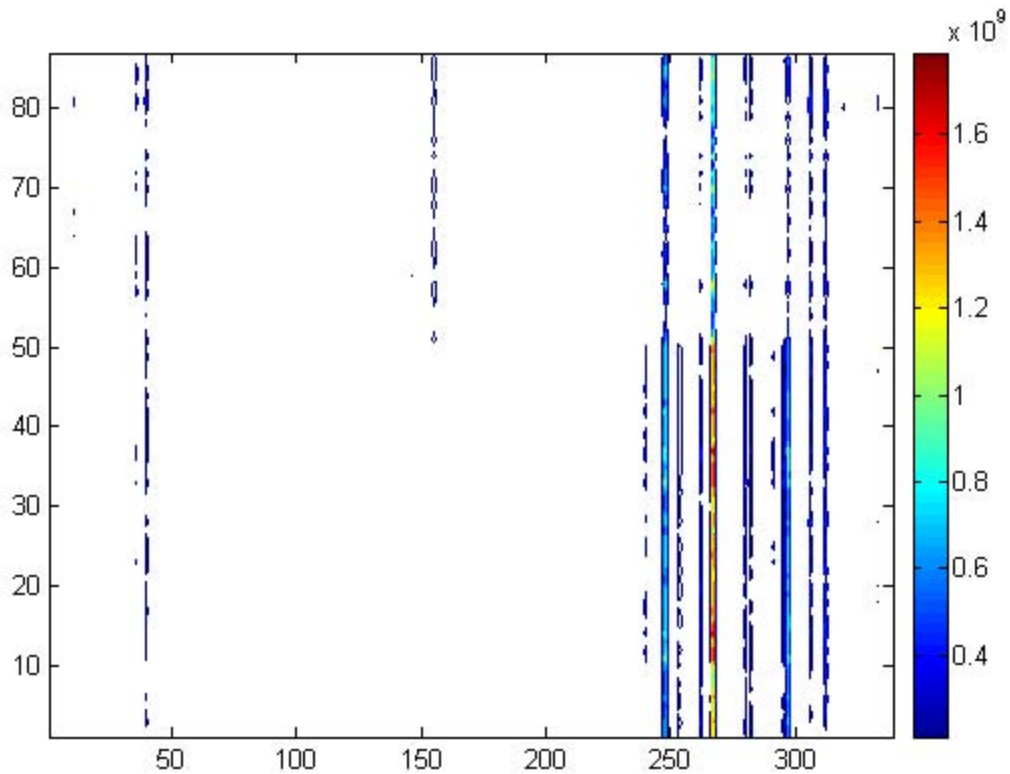

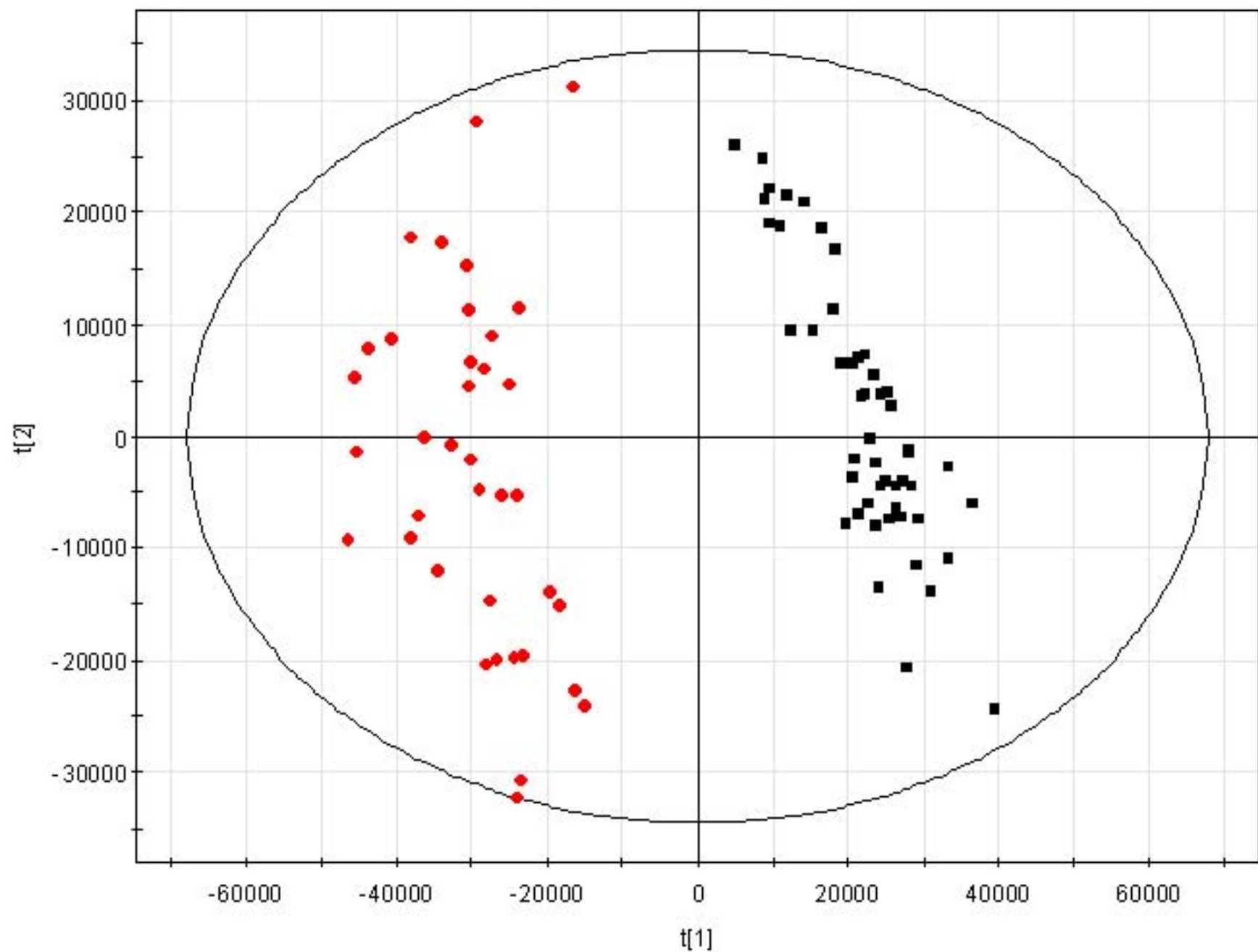

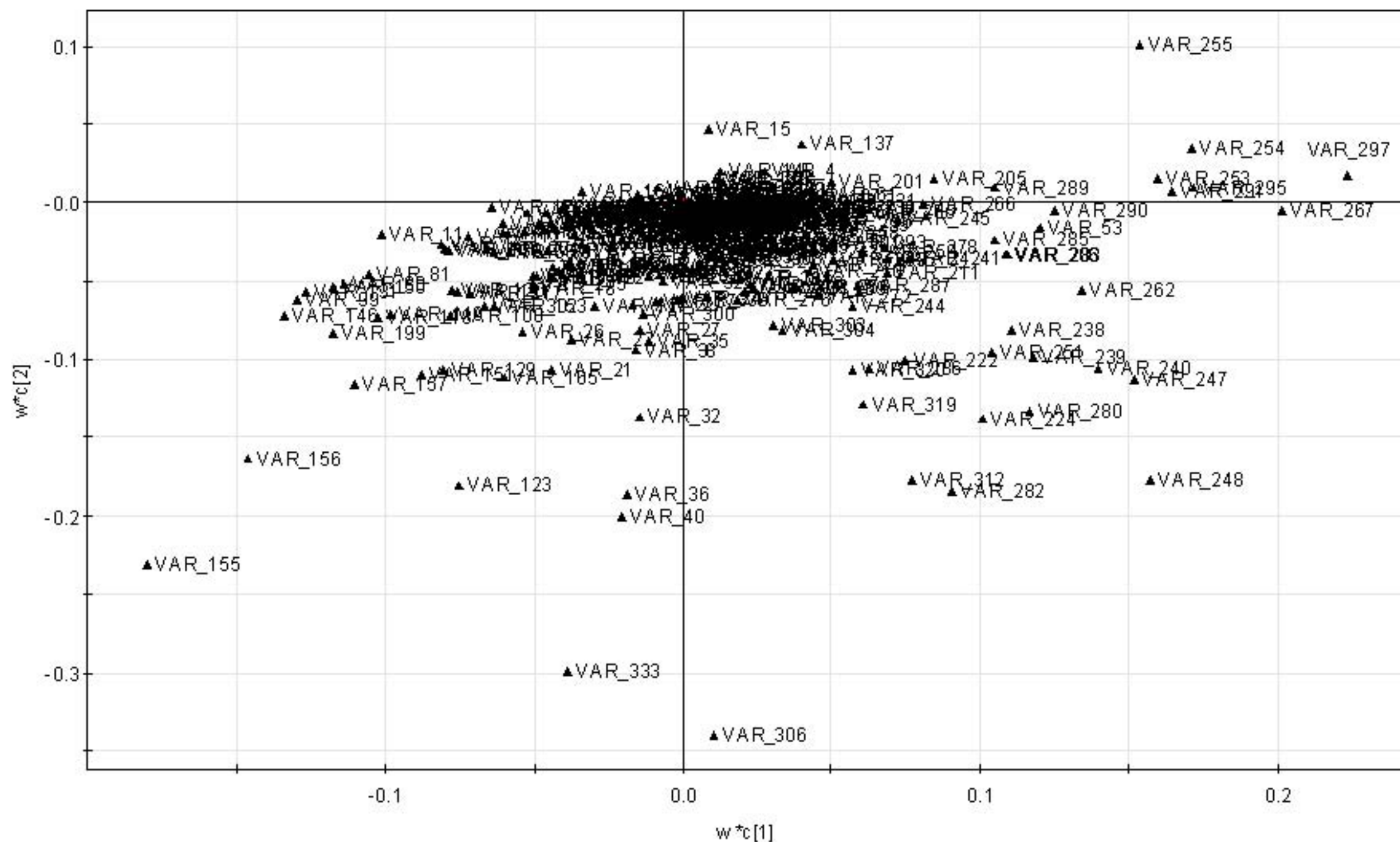

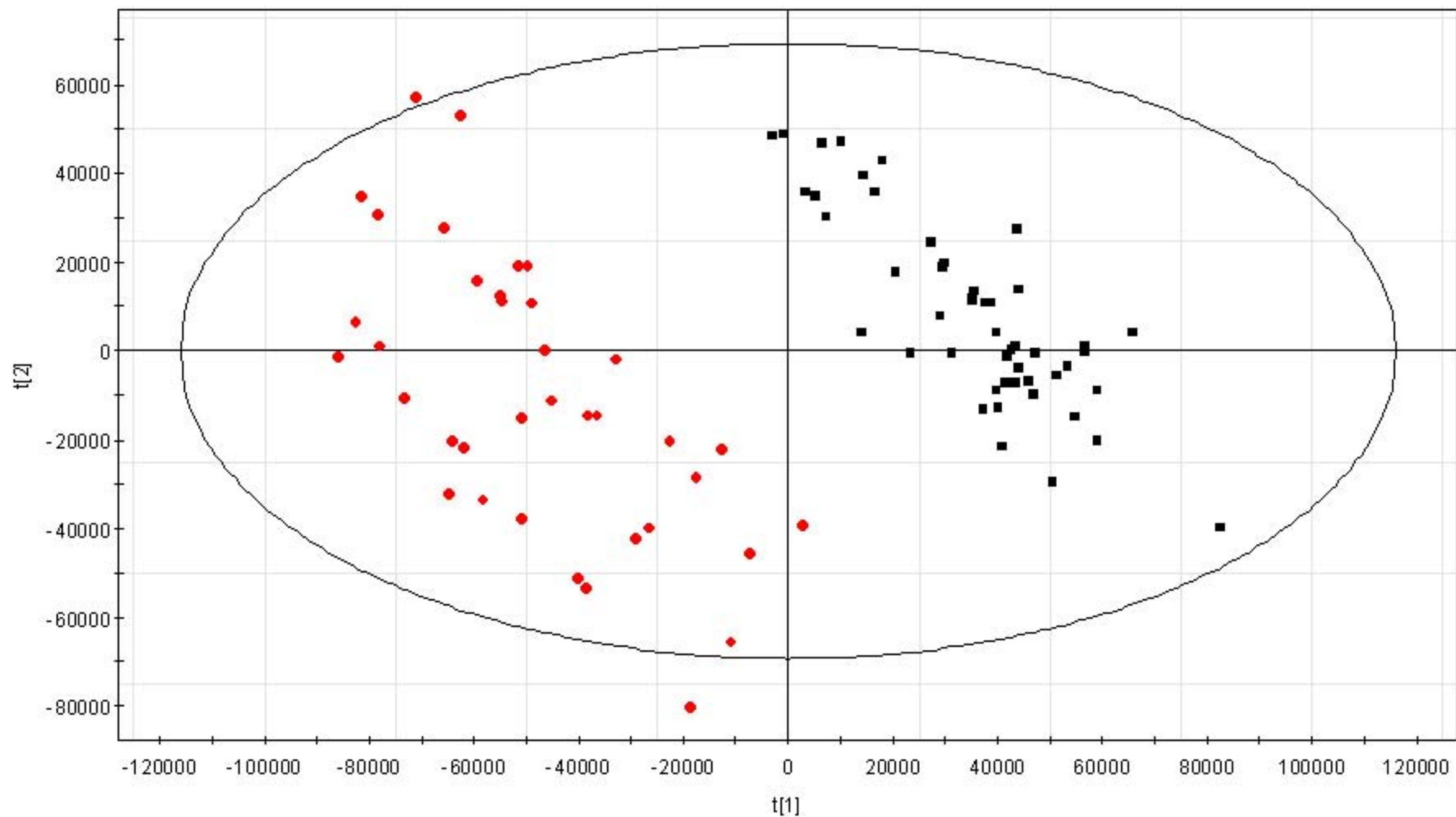

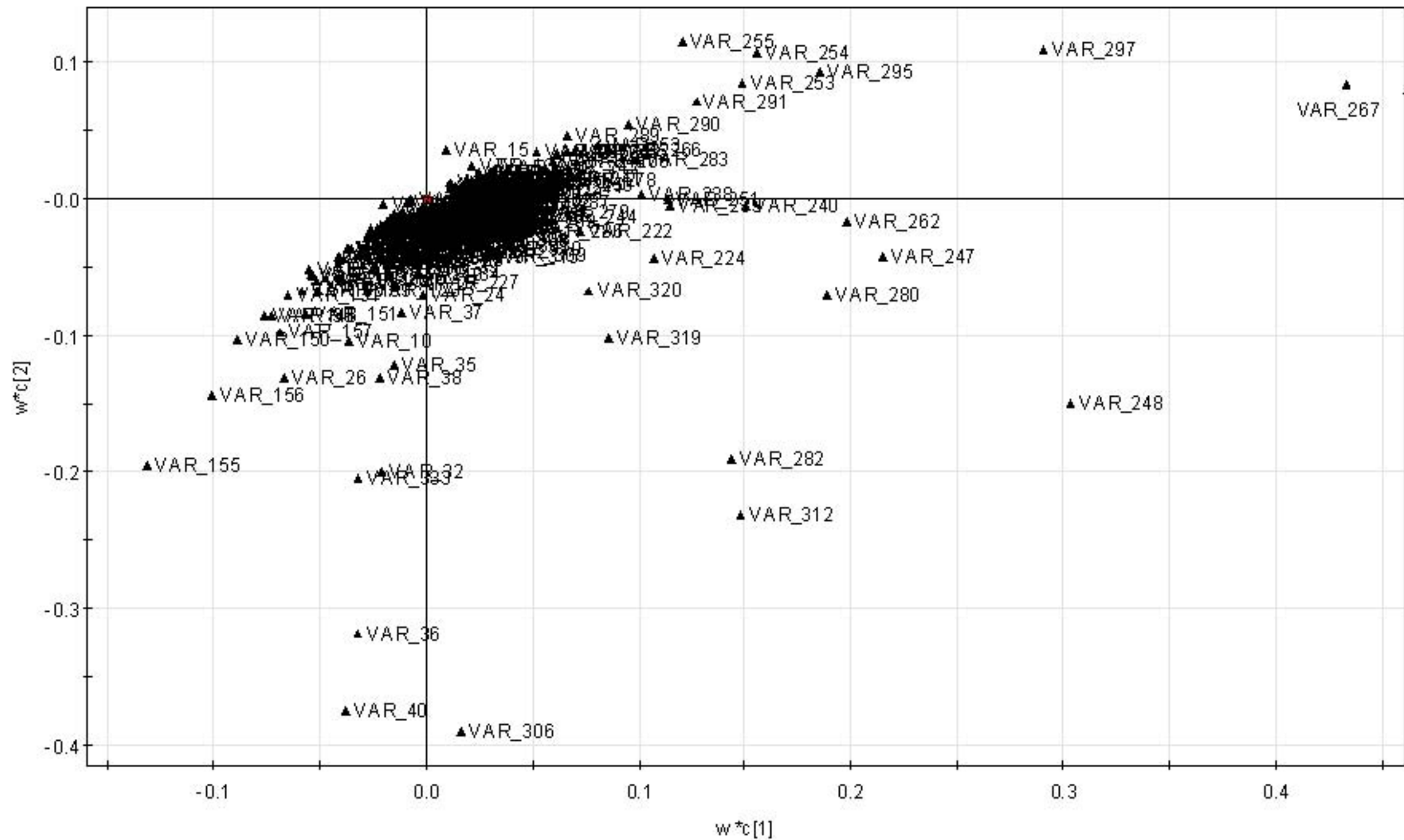

Supplement: Supplementary file 4 [file Image1.PDF]
